# Impact of Regional Hepatic Immunosuppression With Budesonide on Bacterial Elimination in Porcine Abdominal Sepsis

**DOI:** 10.1111/aas.70312

**Published:** 2026-07-23

**Authors:** Katja Hanslin, Paul Skorup, Frida Wilske, Anders Larsson, Eva Tano, Jan Sjölin, Miklos Lipcsey

**Affiliations:** ^1^ Anesthesiology and Intensive Care, Department of Surgical Sciences Uppsala University Uppsala Sweden; ^2^ Section of Infectious Diseases, Department of Medical Sciences Uppsala University Uppsala Sweden; ^3^ Section of Clinical Chemistry, Department of Medical Sciences Uppsala University Uppsala Sweden; ^4^ Section of Clinical Bacteriology, Department of Medical Sciences Uppsala University Uppsala Sweden; ^5^ Hedenstierna Laboratory, Department of Surgical Sciences Uppsala University Uppsala Sweden

## Abstract

**Introduction:**

The liver is essential for bacterial elimination, preventing intestinal bacteria from entering the systemic circulation, and for producing inflammatory cytokines. Glucocorticoids have been reported to exert heterogeneous effects on bacterial clearance in sepsis. Using a porcine model of gram‐negative abdominal sepsis, we investigated how portal venous administration of budesonide, a glucocorticoid with extensive hepatic first‐pass metabolism, affects hepatic bacterial and endotoxin elimination as well as the systemic inflammatory response, in comparison with systemic administration and no treatment.

**Methods:**

The Portal Steroid‐Sepsis (Sep‐Port, *n* = 8) and Systemic Steroid‐Sepsis (Sep‐Syst, *n* = 8) groups were administered budesonide in the portal vein or systemically, followed by an *E. coli* infusion for 3 h in the portal vein. The Septic Controls (Sep‐Ctrl, *n* = 8) received saline instead of budesonide. Non‐septic Controls (NSep‐Port, *n* = 3) were treated only with portal budesonide. Portal, arterial, and hepatic venous bacterial counts were analyzed hourly during the bacterial infusion. The levels of endotoxin and inflammatory cytokines were measured.

**Results:**

There was no difference in hepatic/portal venous bacterial count ratios. However, the arterial and hepatic venous bacterial counts were higher in the Sep‐Syst compared to Sep‐Port group (*p* < 0.001 and *p* < 0.01, respectively), while microbiological findings were similar in the Sep‐Port and Sep‐Ctrl groups. Hepatic endotoxin elimination did not differ between the groups. IL‐10 levels were higher in the Sep‐Port compared to the Sep‐Syst group at 1 h (*p* < 0.01), and IL‐6 levels were lower in the Sep‐Port compared to the Sep‐Ctrl after the bacterial infusion (*p* < 0.05).

**Conclusions:**

In this experimental sepsis model, hepatic bacterial elimination was unaffected by portal or systemic budesonide, whereas systemic administration was associated with increased systemic bacterial levels. Endotoxin clearance was unaffected by budesonide. Portal budesonide elicited a more pronounced anti‐inflammatory response compared to systemic administration. These findings suggest that hepatic exposure to budesonide may modulate the inflammatory response while limiting adverse effects on systemic bacterial clearance.

**Editorial Comment:**

Glucocorticoids are widely used in sepsis management, yet their effects on bacterial clearance is incompletely understood. In this porcine model of gram‐negative abdominal sepsis, portal venous delivery of budesonide (which has ~90% hepatic first‐pass metabolism) preserved bacterial elimination and enhanced the early anti‐inflammatory response, without the increase in systemic bacterial levels seen with systemic administration. These results demonstrate that route of delivery as a potentially important determinant of the risk‐benefit balance of glucocorticoid therapy in sepsis, and support further investigation of hepatic‐targeting strategies.

## 1 Introduction

### 1.1 Background

Severe infections can develop into sepsis and septic shock, with activation of both pro‐ and anti‐inflammatory systems [1]. Inflammatory mediators are initially produced at the site of infection [2, 3, 4], but the inflammatory response rapidly extends beyond the primary site of infection, and high levels of pro‐inflammatory mediators are commonly detected in the systemic circulation, where they may cause distant organ dysfunction [5].

Kupffer cells (KCs), the resident macrophages of the liver, constitute a considerable part of the mononuclear phagocyte system (MPS) [6]. Bacteria and bacterial products from the gut enter the liver via the portal vein, where they are phagocytized by KCs, thereby preventing them from entering the systemic circulation [7, 8]. Although extrahepatic bacterial elimination is substantial in pigs [9], the relative importance of different organs may vary with age [10]. We have previously demonstrated that hepatic elimination reduces bacterial inflow from the gut to a considerable extent in this model [11]. Upon exposure to bacterial ligands such as endotoxin, KCs produce pro‐inflammatory cytokines such as tumor necrosis factor (TNF), interleukin‐1 (IL‐1), and interleukin‐6 (IL‐6) [12, 13]. Rather than being a passive bystander in sepsis, the liver is increasingly recognized as an active immunological organ that may contribute to the propagation of systemic inflammation during sepsis [14]. The liver could thus be an important component in triggering the systemic inflammatory response, ultimately leading to multiple organ failure. Consequently, suppression of the hepatic inflammatory response locally could potentially counteract the pathophysiologic changes in sepsis. Glucocorticoids are potent anti‐inflammatory agents that could potentially attenuate the hepatic inflammatory response in sepsis. However, treatment with glucocorticoids has been shown to decrease bacterial clearance [15], thereby increasing the risk of infectious complications, particularly with prolonged use [16]. The balance between the anti‐inflammatory effects of glucocorticoids and their effects on bacterial clearance is not fully characterized.

We hypothesized that using a glucocorticoid with extensive first‐pass metabolism in the liver could allow regional immunomodulation within the liver while minimizing systemic effects on bacterial clearance. Budesonide undergoes approximately 90% first‐pass hepatic metabolism, resulting in low systemic availability [17, 18, 19]. Accordingly, using an experimental porcine model of sepsis, we set out to investigate whether targeted hepatic administration of budesonide could modulate the inflammatory response to a bacterial challenge without compromising hepatic bacterial and endotoxin elimination or systemic bacterial clearance, compared with systemic glucocorticoid administration.

### 1.2 Objectives

The primary objective was to investigate whether targeted administration of budesonide in the liver alters hepatic bacterial elimination compared with systemic administration in an experimental sepsis model. Secondary objectives were to examine the effects on systemic bacterial clearance, hepatic endotoxin elimination, and inflammatory and physiological responses, thereby assessing whether regional hepatic immunomodulation can be achieved without impairing bacterial and endotoxin clearance.

## 2 Material and Methods

### 2.1 Ethical Statement

The Animal Ethics Board in Uppsala, Sweden, approved the experiment (Dnr. C155/14 and 5.8.18‐08592‐/2019). The pigs were handled in accordance with the Guide for the Care and Use of Laboratory Animals (EU Directive 2010/63/EU). Minimum Quality Threshold in Preclinical Sepsis Studies (MQTiPSS) guidelines were followed when relevant for these experiments [20], and ARRIVE guidelines [21, 22] were followed when reporting.

### 2.2 Study Design

**FIGURE 1:**
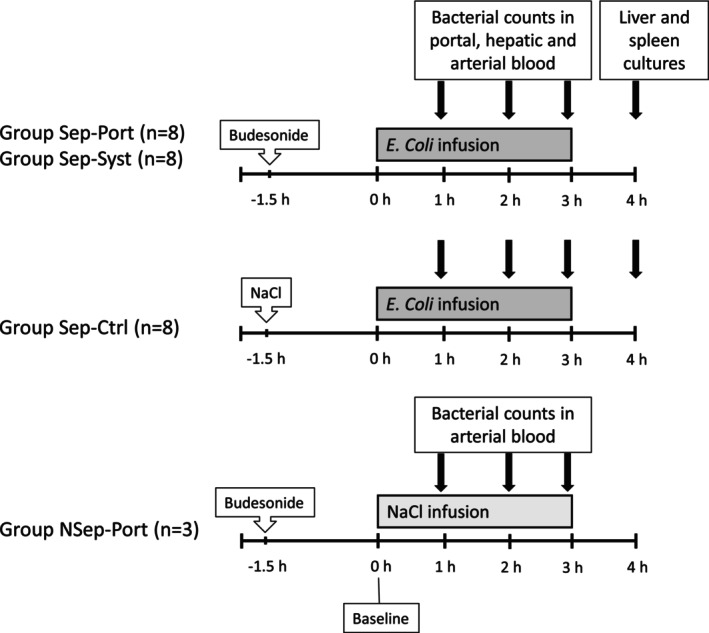
Study design. The Portal Steroid‐Sepsis (Sep‐Port) group received budesonide via the portal vein, while the Systemic Steroid‐Sepsis (Sep‐Syst) group received budesonide via a central vein. Animals in the Septic Control (Sep‐Ctrl) group received saline (NaCl) instead of budesonide. All animals in the Sep‐Port, Sep‐Syst, and Sep‐Ctrl groups were subjected to an *E. coli* infusion for 3 h, starting at 0 h, 90 min after initiation of the budesonide or saline infusion. The Non‐septic Control (NSep‐Port) group received budesonide via the portal vein, followed by an infusion of saline instead of *E. coli* . Baseline indicates the start of the bacterial or saline infusion in all groups.

A total of twenty‐seven pigs were included in the study. The study design is illustrated in Figure 1. The animals were allocated to one of four groups: The Portal Steroid‐Sepsis (Sep‐Port, *n* = 8), the Systemic Steroid‐Sepsis (Sep‐Syst, *n* = 8), the Septic Controls (Sep‐Ctrl, *n* = 8) and the Non‐septic Controls (NSep‐Port, *n* = 3).

### 2.3 Experimental Procedures

The experiments were carried out in an intensive care unit (ICU)‐like setting in an animal research facility with experienced staff. The animals were treated according to a protocol to maintain vital parameters within pre‐set limits (Table M1 in the Supporting Information). Detailed information on the preparatory procedure and information on anesthesia and fluid administration can also be found in the Supporting Information. All animals were anesthetized, mechanically ventilated throughout the experiment, and catheterized with an arterial line, a central venous catheter, a Swan‐Ganz catheter and a hepatic venous catheter. Two catheters were placed in the portal vein (Figure M1 in Supporting Information). A proximal portal venous catheter, defined as positioned a few centimeters from the liver, was used for blood sampling. A distal portal venous catheter, positioned approximately 2 cm upstream (i.e., further from the liver) relative to the proximal catheter, was used for portal infusion of *E. coli* and budesonide (the Sep‐Port group). Thus, blood sampling for bacterial cultures was performed downstream of the infusion site, immediately before entering the liver. After preparations, 25 mg budesonide (Budesonid, Teva Pharma B.V. Haarlem, the Netherlands) in 50 mL saline was administered in 15 min through the distal portal venous catheter in groups Sep‐Port and NSep‐Port and through the central venous catheter in the Sep‐Syst group. The animals in Sep‐Ctrl group received saline intravenously instead of budesonide. To mimic bacterial influx from the gut, all animals in the Sep‐Port, Sep‐Syst and Sep‐Ctrl groups were exposed to a continuous infusion of live *Escherichia coli (E. coli), a common gut pathogen*, in the portal vein through the distal portal venous catheter for 3 h. Bacteria were grown into logarithmic growth phase before the experiments, and the *E. coli* infusion was initiated 90 min after completion of the budesonide infusion. To study the effects of portal budesonide alone on the animals, the animals in NSep‐Port group received an infusion of saline, instead of *E. coli* . In the Sep‐Port, Sep‐Syst, and Sep‐Ctrl groups, simultaneous blood samples for bacterial counts were obtained hourly during the *E. coli* infusion from the proximal portal venous catheter, downstream of the *E. coli* infusion site, as well as from the artery and the hepatic vein. Arterial blood was analyzed for bacterial counts 15 and 30 min after completion of the *E. coli* infusion. In the NSep‐Port group, cultures from arterial blood were collected every hour. A detailed description of the organism and bacteriological methods can be found in the Supporting Information. At the end of the experiment, the animals were sacrificed by injection of 20 mL potassium chloride. Samples from the liver and spleen were obtained for bacterial cultures, and liver samples were collected for histopathological analysis.

### 2.4 Measurements

All physiological data were registered every hour starting at baseline. Circulatory and respiratory variables, as well as creatinine clearance during the experiment, were calculated using standard formulae [23]. Plasma endotoxin levels were measured in heparinized blood samples obtained from the proximal portal vein, the hepatic vein, and the artery at baseline (0 h, prior to initiation of the bacterial infusion) and at 3 h, using a chromogenic limulus amebocyte lysate assay (Endochrome K; Charles River Endosafe, Charleston, SC, USA), with a lower detection limit of < 0.05 EU × mL^−1^. Blood samples for analysis of TNF, IL‐6, IL‐10, blood gasses, full blood count, and creatinine were obtained from arterial blood before administering budesonide and then hourly. Plasma TNF, IL‐6, and IL‐10 concentrations were measured using porcine specific sandwich enzyme linked immunosorbent assays (ELISA; R&D Systems, Minneapolis, MN, USA), with limits of detection of < 60 pg × mL^−1^ for TNF, and IL‐6 and 20 pg × mL^−1^ for IL‐10. The assays exhibited total coefficients of variation of approximately 6%. Full blood counts were analyzed using an automated hematology analyzer (Sysmex XN; Sysmex, Kobe, Japan). Urine output was measured continuously, and urine samples for creatinine analysis were collected at predetermined intervals. Creatinine in plasma and urine were analyzed on a Mindray BS430 chemistry analyzer (Mindray Medical International, Shenzhen, China) using IDMS traceable enzymatic creatinine reagents from Abbott Laboratories (Abbott Park, IL, USA). A more detailed description of the measurements, blood and urine sampling, and histopathological analyses is provided in the Supporting Information.

### 2.5 Endpoints

The primary endpoint was hepatic bacterial elimination following portal versus systemic or no budesonide administration, assessed as the hepatic to portal venous bacterial count ratios. Secondary endpoints included systemic bacterial levels measured as arterial bacterial counts and hepatic endotoxin elimination, assessed as the hepatic to portal venous endotoxin ratios. The inflammatory response was assessed by measuring systemic cytokine levels (TNF, IL‐6, and IL‐10), and the physiological responses were evaluated by recording and comparing respiratory, circulatory, and metabolic variables.

### 2.6 Experimental Animals

Juvenile Norwegian Landrace pigs (8–10 weeks old) of both sexes were used for the experiments. This age was chosen because the animals are pre‐pubertal, allowing inclusion of both sexes, and their size enables advanced ICU‐style instrumentation, continuous monitoring, and repeated blood sampling. Detailed information on housing can be found in the Supporting Information.

### 2.7 Calculations and Statistical Methods

The statistical analysis was planned prior to study initiation and performed accordingly. Based on an assumed standard deviation of 0.20 for the primary endpoint, a sample size of seven animals per group provided 80% power to detect a 30% difference (two‐sided *α* = 0.05). Bacterial counts were adjusted for variations in animal size and infused bacterial dose. Data were tested for normality, and data with a log‐normal distribution were log‐transformed. Data are presented as mean ± SD for normally distributed variables and as median (interquartile range) for non‐normally distributed variables. For normally distributed data, Student's *t‐*test was used for comparisons between groups. ANOVA III for repeated measurements was used to assess group differences and changes over time. For non‐normally distributed data, Mann–Whitney *U* test was performed for intergroup comparisons. Correlations were analyzed by the Spearman rank test. All analyses were performed using Statistica software (version 14, StatSoft Inc., Tulsa, OK, USA) and a *p*‐value of < 0.05 was considered significant. The NSep‐Port group was not included in the statistical analysis.

## 3 Results

### 3.1 Baseline Data

The baseline variables were similar in the Sep‐Port, Sep‐Syst, and Sep‐Ctrl groups (Table 1). All animals survived throughout the experiment. Sepsis, according to Sepsis‐3 criteria [24], developed in all animals in the Sep‐Ctrl group, compared with three in the Sep‐Port group and none in the Sep‐Syst group.

**TABLE 1:** Animals' weight and physiological variables at baseline and during the experiment.

Variable	Sep‐Port (*n* = 8)	Sep‐Syst (*n* = 8)	Sep‐Ctrl (*n* = 8)	NSep‐Port (*n* = 3)
Weight (kg)	25.7 ± 0.9	25.4 ± 1.8	25.4 ± 1.5	26.8 ± 0.3
PaO_2_/FiO_2_ ratio (kPa)				
0 h	62 ± 6	63 ± 3	63 ± 4	67 ± 9
1 h	56 ± 8*^,††^	57 ± 7	48 ± 9	61 ± 10
2 h	50 ± 8*^,††^	54 ± 6	47 ± 6	56 ± 5
3 h	47 ± 11*^,††^	54 ± 5	39 ± 6	54 ± 5
4 h	47 ± 10*^,††^	54 ± 6	39 ± 7	48 ± 7
Static compliance (mL × cmH_2_O^−1^)				
0 h	36.8 ± 5.6	35.8 ± 5.5	35.1 ± 9.1	53.6 ± 13.1
1 h	31.3 ± 7.6^††^	30.9 ± 3.8	27.0 ± 7.2	42.6 ± 7.6
2 h	27.0 ± 5.5^††^	29.0 ± 3.5	22.0 ± 5.7	40.8 ± 6.5
3 h	26.1 ± 5.9^††^	28.8 ± 4.2	20.5 ± 4.5	41.2 ± 6.7
4 h	25.6 ± 6.1^††^	26.6 ± 3.3	20.9 ± 4.7	37.5 ± 11.0
Mean arterial pressure (mmHg)				
0 h	90 ± 9	96 ± 9	88 ± 9	77 ± 14
1 h	86 ± 15***^,†††^	100 ± 15	84 ± 12	77 ± 7
2 h	105 ± 15***^,†††^	124 ± 12	95 ± 14	77 ± 8
3 h	99 ± 17***^,†††^	120 ± 13	78 ± 14	76 ± 2
4 h	94 ± 17***^,†††^	115 ± 15	81 ± 16	71 ± 11
Mean pulmonary arterial pressure (mmHg)				
0 h	18 ± 5	21 ± 4	17 ± 3	22 ± 3
1 h	23 ± 7^†^	25 ± 7	32 ± 3	22 ± 4
2 h	33 ± 9^†^	31 ± 7	34 ± 4	22 ± 6
3 h	32 ± 7^†^	31 ± 7	33 ± 8	20 ± 3
4 h	25 ± 5^†^	26 ± 8	29 ± 8	21 ± 2
Cardiac index (L × min‐^1^ × m‐^2^)				
0 h	3.1 ± 0.5	2.7 ± 0.3	2.5 ± 0.5	3.0 ± 0.6
1 h	3.0 ± 0.5^†††^	3.1 ± 0.6	2.5 ± 0.6	3.0 ± 0.7
2 h	2.7 ± 0.5^†††^	2.4 ± 0.3	2.3 ± 0.3	3.0 ± 0.3
3 h	2.3 ± 0.5^†††^	2.2 ± 0.3	1.9 ± 0.4	3.1 ± 0.4
4 h	2.5 ± 0.2^†††^	2.5 ± 0.4	2.1 ± 0.2	3.4 ± 0.5
Arterial lactate (mmol × L^−1^)				
0 h	2.1 (1.9–2.3)	2.0 (1.8–2.8)	2.4 (1.8–2.7)	1.6 (1.5–3.4)
1 h	2.3 (2.2–2.4)^†††^	1.9 (1.7–2.9)	2.8 (2.1–3.7)	1.6 (1.4–3.0)
2 h	1.9 (1.8–2.2)^†††^	1.8 (1.6–2.2)	2.3 (1.8–2.8)	1.3 (1.3–1.9)
3 h	1.7 (1.5–2.1)^†††^	1.6 (1.4–1.7)	2.6 (2.1–3.5)	1.1 (1.1–1.5)
4 h	1.1 (1.1–1.2)^†††^	1.1 (1.0–1.3)	1.9 (1.3–2.4)	1.0 (0.9–1.2)
Base excess (mmol × L^−1^)				
0 h	5.2 (4.9–6.6)	5.8 (5.0–6.3)	6.2 (4.5–6.6)	6.4 (5.8–6.6)
1 h	4.9 (3.8–5.2)^†^	5.3 (3.6–5.8)	3.5 (1.8–5.0)	6.5 (3.8–7.2)
2 h	4.8 (4.2–5.3)^†^	5.6 (4.3–6.3)	3.9 (2.9–5.6)	6.7 (4.7–6.9)
3 h	5.2 (3.7–5.8)^†^	5.9 (4.5–7.9)	2.7 (2.0–5.2)	7.1 (6.8–7.7)
4 h	6.5 (5.8–7.2)^†^	7.8 (5.9–9.2)	4.8 (3.5–6.4)	7.1 (6.5–7.9)
Arterial glucose (mmol × L^−1^)				
0 h	8.6 (8.1–9.6)	8.4 (8.2–10.2)	8.5 (7.6–10.0)	8.0 (7.0–9.5)
1 h	8.6 (8.0–9.1)**^,††^	9.2 (8.3–10.2)	7.4 (7.0–8.9)	7.6 (6.9–9.1)
2 h	7.5 (6.6–7.9)**^,††^	7.6 (7.1–8.4)	6.1 (5.9–6.5)	7.4 (7.1–8.7)
3 h	6.7 (6.2–7.4)**^,††^	7.3 (6.8–7.7)	5.8 (5.5–6.2)	7.4 (7.1–8.4)
4 h	6.1 (5.7–6.3)**^,††^	6.3 (5.9–7.0)	5.4 (5.4–5.7)	7.2 (6.9–8.0)
Hemoglobin (g × L^−1^)				
0 h	83 ± 5	82 ± 6	79 ± 6	75 ± 3
1 h	86 ± 4	84 ± 6	88 ± 6	72 ± 4
2 h	90 ± 4	87 ± 5	93 ± 7	69 ± 4
3 h	93 ± 5	90 ± 5	96 ± 12	67 ± 3
4 h	87 ± 6	87 ± 7	93 ± 16	66 ± 6
Leukocyte count (× 10^9^ × L^−1^)				
0 h	23.4 (21–26.8)	19.6 (16.6–24.5)	15.6 (12.5–18.9)	19.9 (13.1–25.0)
1 h	9.48 (6.8–19.4)^†††^	9.4 (5.0–11.0)	7.2 (6.2–7.4)	19.5 (15.5–39.3)
2 h	12.3 (6.8–22.8)^†††^	11.6 (8.3–18.3)	6.6 (4.2–7.8)	19.8 (15.8–42.0)
3 h	10.3 (7.5–26.4)^†††^	14.1 (11.6–19.7)	6.1 (5.2–9.6)	19.1 (14.8–39.8)
4 h	10.1 (8.0–27.8)^†††^	18.7 (14.5–22.1)	6.4 (5.4–10.1)	20.0 (13.2–37.3)
Neutrophil granulocyte count (× 10^9^ × L^−1^)				
0 h	16.6 (15.2–18.9)	13.3 (11.1–19.0)	6.5 (5.4–11.0)	12.0 (8.2–19.3)
1 h	5.5 (4.2–16.1)^†††^	6.8 (2.5–8.5)	2.2 (1.2–3.7)	12.6 (11.6–33.7)
2 h	9.0 (4.5–20.4)^†††^	9.5 (6.0–16.1)	2.6 (1.5–3.6)	13.0 (12.2–36.4)
3 h	7.3 (5.2–23.6)^†††^	12.0 (9.217.4)	2.8 (1.9–5.3)	12.8 (11.1–34.6)
4 h	7.5 (5.7–24.6)^†††^	15.9 (12.0–19.7)	3.5 (2.8–6.7)	13.3 (9.5–31.8)
Temperature (°C)				
0 h	39.4 ± 1.0	38.9 ± 1.1	38.8 ± 0.9	38.8 ± 1.0
1 h	39.8 ± 1.1	39.6 ± 1.3	39.5 ± 1.2	39.6 ± 1.0
2 h	40.5 ± 1.2	40.4 ± 1.4	40.0 ± 1.5	40.3 ± 1.2
3 h	40.9 ± 1.1	40.6 ± 1.2	40.7 ± 1.5	40.6 ± 0.9
4 h	41.1 ± 0.9	40.5 ± 0.9	40.9 ± 1.6	41.1 ± 1.0
Urine output (mL × kg^−1^ × h^−1^)				
Before baseline	1.8 (1.2–2.4)	1.7 (0.8–5.3)	2.6 (1.4–3.9)	3.6 (1.0–8.8)
0–4 h	4.9 (3.2–6.7)**	8.4 (6.4–9.6)	4.9 (3.1–6.7)	5.6 (3.6–6.7)
Creatinine clearance (mL × min^−1^)				
Before baseline	99 (75–104)	94 (50–109)	105 (69–116)	95 (65–167)
0–4 h	99 (89–113)^††^	108 (98–116)	78 (66–93)	91 (89–111)

*Note:* Normally distributed data are presented as mean ± SD, whereas non‐normally distributed data as median (IQR). Data were log‐transformed when appropriate for the statistical analysis, but all data are presented in non‐log form. The differences between the Portal Steroid‐Sepsis (Sep‐Port) and the Systemic Steroid‐Sepsis (Sep‐Syst), and the Sep‐Port and the Septic Controls (Sep‐Ctrl) groups 1–4 h were assessed using ANOVA III for repeated measurements. *p < 0.05, **p < 0.01, ***p < 0.001 for differences between Sep‐Port and Sep‐Syst groups; †*p* < 0.05, ††*p* < 0.01, †††*p* < 0.001 for differences between Sep‐Port and Sep‐Ctrl groups. The Sep‐Port and Sep‐Syst groups were administered budesonide in the portal vein or systemically, followed by an *E. coli* infusion for 3 h in the portal vein. The Sep‐Ctrl received saline instead of budesonide. Non‐septic Controls (NSep‐Port, *n* = 3) were treated only with portal budesonide.

### 3.2 Hepatic Bacterial Elimination

**FIGURE 2:**
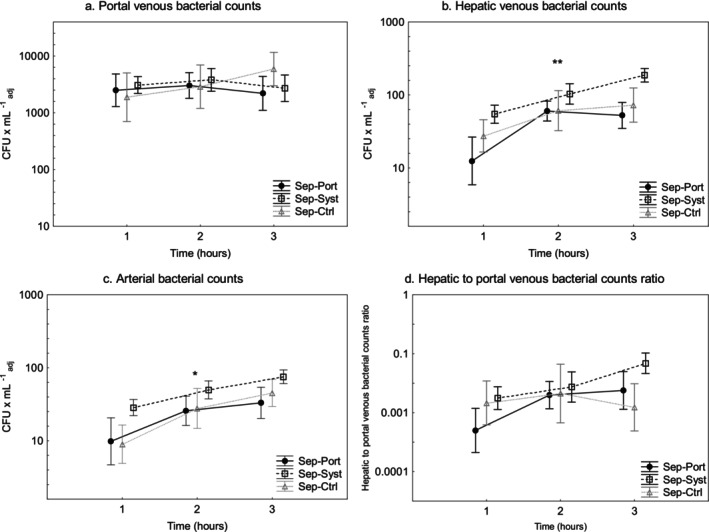
Hepatic bacterial elimination, assessed as the hepatic to portal venous bacterial count ratios, was similar between groups. Arterial and hepatic venous bacterial counts were higher following systemic budesonide administration compared with portal budesonide administration. Bacterial counts in the portal vein, hepatic vein and artery during the *E. coli* infusion (a–c). The ratio of hepatic to portal venous bacterial counts during the *E. coli* infusion (d). The Sep‐Port (*n* = 8) and Sep‐Syst (*n* = 8) groups received budesonide via the portal vein or systemically, respectively, followed by a portal *E. coli* infusion for 3 h. The Sep‐Ctrl group (*n* = 8) received saline instead of budesonide. Values are expressed as mean ± SEM. *p < 0.05, **p < 0.01, denote differences between the Sep‐Port and Sep‐Syst groups. Differences between the Sep‐Port and Sep‐Syst groups, and between the Sep‐Port and Sep‐Ctrl groups, were assessed using ANOVA III for repeated measurements.

The doses of administered bacteria were comparable between the Sep‐Port, Sep‐Syst, and Sep‐Ctrl groups (4.29 ± 0.06, 4.29 ± 0.06, and 4.28 ± 0.09 log_10_ CFU × g^−1^, respectively). There was no growth of bacteria in the blood in the NSep‐Port group during the experiment. Portal venous bacterial counts were similar between the groups. However, the bacterial counts in arterial and hepatic venous blood were higher in the Sep‐Syst compared to the Sep‐Port group (*p* < 0.05 and *p* < 0.01, respectively; Figure 2). Hepatic to portal venous bacterial count ratios were similar between the Sep‐Port and Sep‐Syst groups, and there were no differences in bacterial counts or bacterial count ratios between the Sep‐Port and Sep‐Ctrl groups (Figure 2).

### 3.3 Post‐Infusion Bacterial Concentrations

Circulating bacteria were rapidly cleared after completion of the *E. coli* infusion, with no differences between groups at either time point. At 15 min, median (IQR) bacterial counts were 0 (0–10), 0 (0–7), and 0 (0–3) CFU × mL^−1^ in the Sep‐Port, Sep‐Syst, and Sep‐Ctrl groups, and at 30 min, counts were 0 (0–0), 0 (0–0), and 0 (0–2) CFU × mL^−1^, respectively.

### 3.4 Growth of Bacteria in the Liver and the Spleen

**FIGURE 3:**
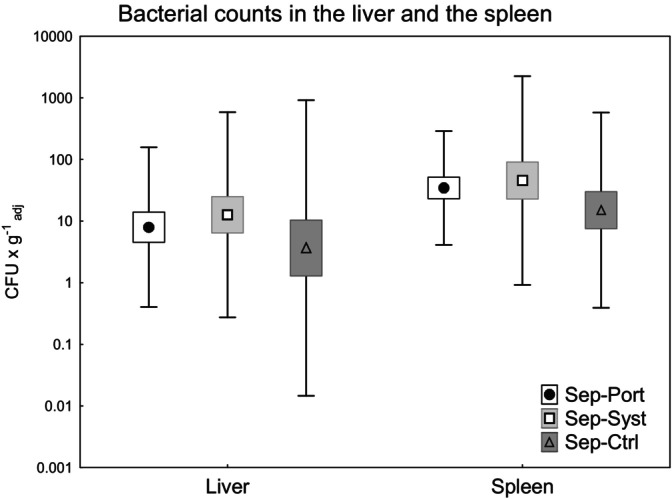
Bacterial growth in the liver and spleen did not differ between groups. Bacterial counts in the liver and spleen in the Sep‐Port (*n* = 7), Sep‐Syst (*n* = 8), and Sep‐Ctrl (*n* = 7) groups. Sep‐Port and Sep‐Syst animals received budesonide via the portal vein or systemically, respectively, followed by a 3‐h portal *E. coli* infusion. Sep‐Ctrl animals received saline instead of budesonide. Bacterial counts were adjusted for variations in animal size and infused bacterial dose. Data are presented with the central marker indicating the mean, boxes representing mean ± SE, and whiskers indicating ± SD. Differences between the Sep‐Port and Sep‐Syst groups, and between the Sep‐Port and Sep‐Ctrl groups, were assessed using Student's *t*‐test.

There was no difference in the growth of *E. coli* in the liver or spleen between the Sep‐Port and Sep‐Syst groups, or the Sep‐Port and Sep‐Ctrl groups (Figure 3). Bacterial growth in the liver and spleen could not be determined in two animals (one Sep‐Port and one Sep‐Ctrl) due to a technical error.

### 3.5 Hepatic Endotoxin Elimination

Before the start of the bacterial infusion, endotoxin levels were low, mostly below the detection limit, in blood at all sites (Table 2). Portal venous, hepatic venous, and arterial blood endotoxin levels, as well as hepatic to portal venous endotoxin ratios at 3 h, were similar between the Sep‐Port and Sep‐Syst groups and between the Sep‐Port and Sep‐Ctrl groups.

**TABLE 2:** Endotoxin levels in the portal vein, hepatic vein and the artery at 0 h, before the start of the *E. coli* infusion, and at 3 h during the *E. coli* infusion.

	Time	Sep‐Port (*n* = 8) EU × mL^−1^	Sep‐Syst (*n* = 8) EU × mL^−1^	Sep‐Ctrl (*n* = 8) EU × mL^−1^
Portal vein	0 h	0.05 (< 0.05–0.09)	< 0.05 (< 0.05–0.09)	0.05 (< 0.05–0.08)
	3 h	6.3 (4.3–7.5)	7.8 (3.8–11.3)	18.3 (6.8–28.0)
Hepatic vein	0 h	0.05 (< 0.05–0.08)	0.13 (0.06–0.22)	0.10 (< 0.05–0.43)
	3 h	3.1 (2.0–4‐4)	2.4 (2.1–4.1)	3.5 (2.4–5.0)
Artery	0 h	< 0.05 (< 0.05‐ < 0.05)	< 0.05 (< 0.05–0.09)	< 0.05 (< 0.05‐ < 0.05)
	3 h	3.0 (1.6–4.4)	2.1 (1.9–4.1)	3.5 (2.2–4.8)
Hepatic to portal venous endotoxin ratio	3 h	0.52 (0.47–0.68)	0.50 (0.26–0.80)	0.20 (0.12–0.70)

*Note:* Data were non‐normally distributed and are presented as median (IQR). The Sep‐Port and Sep‐Syst groups were administered budesonide in the portal vein or systemically, and thereafter an *E. coli* infusion for 3 h in the portal vein. The Sep‐Ctrl received saline instead of budesonide. The difference between the Portal Steroid‐Sepsis (Sep‐Port) and the Systemic Steroid‐Sepsis (Sep‐Syst), and Sep‐Port and the Septic Controls (Sep‐Ctrl) groups at 0 h and 3 h was assessed using the Mann–Whitney *U* test. There were no differences in endotoxin levels or in the hepatic to portal venous endotoxin ratios between the Sep‐Port and Sep‐Syst, or the Sep‐Port and Sep‐Ctrl groups.

### 3.6 Cytokine Response

**FIGURE 4:**
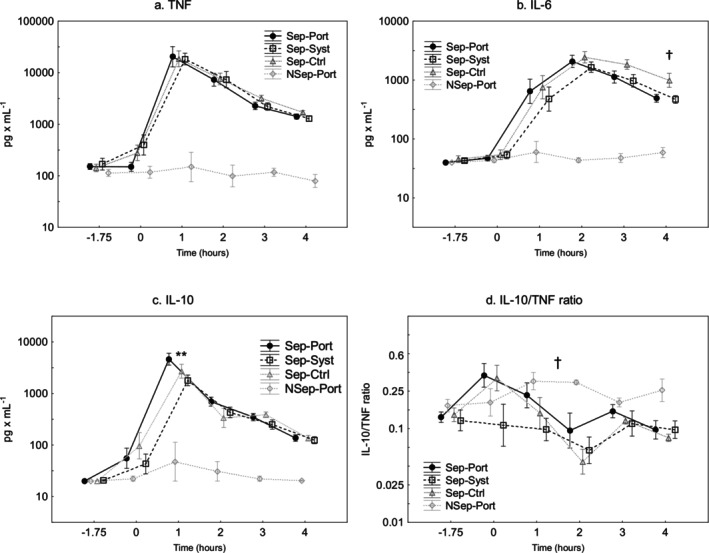
Peak IL‐6 levels were lower in the Sep‐Port group compared with the Sep‐Ctrl group at 4 h (post hoc, *p* < 0.05), and peak IL‐10 levels were higher in the Sep‐Port than in the Sep‐Syst group (*p* < 0.01). The IL‐10/TNF ratio was also higher in the Sep‐Port compared with the Sep‐Ctrl group (*p* < 0.05). The levels of tumor necrosis factor (TNF) (a), interleukin‐6 (IL‐6) (b), interleukin‐10 (IL‐10) (c), and the IL‐10/TNF ratio (d) during the experiment. Sep‐Port and Sep‐Syst animals received budesonide via the portal vein or systemically, respectively, followed by a 3‐h portal *E. coli* infusion. Sep‐Ctrl animals received saline instead of budesonide. Values are expressed as mean ± SEM. *p < 0.01 denotes differences between the Sep‐Port and Sep‐Syst groups; †*p* < 0.05 denotes differences between the Sep‐Port and the Sep‐Ctrl groups. All assessed using ANOVA III for repeated measurements.

TNF levels peaked after 1 h, with no differences between groups during the experiment (Figure 4). IL‐6 levels peaked after 2 h in all groups, with lower levels in the Sep‐Port group vs. the Sep‐Ctrl group at 4 h in a post hoc analysis (*p* < 0.05). IL‐10 levels peaked at 1 h and were higher in the Sep‐Port than in the Sep‐Syst group (*p* < 0.01), without difference between the Sep‐Port and Sep‐Ctrl groups. The IL‐10/TNF ratio was higher in the Sep‐Port vs. Sep‐Ctrl group (*p* < 0.05), with no other between‐group differences.

### 3.7 Physiological Responses

The PaO_2_/FiO_2_ ratio decreased in all groups during the bacterial infusion and was lower in the Sep‐Port compared with the Sep‐Syst group (*p* < 0.05), but higher compared with the Sep‐Ctrl group (*p* < 0.01, Table 1). Static compliance was higher in the Sep‐Port vs. Sep‐Ctrl group (*p* < 0.01). Mean arterial pressure (MAP) was lower in the Sep‐Port vs. Sep‐Syst group (*p* < 0.001), but higher compared to the Sep‐Ctrl group (*p* < 0.001). Noradrenaline was administered in one pig in the Sep‐Port group, in no pigs in the Sep‐Syst group, and in five pigs in the Sep‐Ctrl group. The total cumulative noradrenaline dose was 0 (0–0.23) μg × kg^−1^ in the Sep‐Port group, none in the Sep‐Syst group, and 4.85 (0–18.19) μg × kg^−1^ in the Sep‐Ctrl group (median (IQR)), with no differences between the Sep‐Port and Sep‐Syst, or the Sep‐Port and Sep‐Ctrl groups. The total volume of intravenous fluids administered, in addition to maintenance fluids, was 0 (0–7) in the Sep‐Port group, 0 (0–8) in the Sep‐Syst group, and 15 (0–22) in the Sep‐Ctrl group (median (IQR), mL × kg^−1^), with no differences observed between the Sep‐Port and Sep‐Syst groups or between the Sep‐Port and Sep‐Ctrl groups. Mean pulmonary arterial pressure (MPAP) was lower and cardiac index (CI) was higher in the Sep‐Port compared with the Sep‐Ctrl group at 1–4 h (*p* < 0.05 and *p* < 0.001, respectively). Arterial lactate levels were lower and base excess levels were higher in the Sep‐Port compared with the Sep‐Ctrl group (*p* < 0.001 and p < 0.05, respectively). Arterial glucose levels were lower in the Sep‐Port compared with the Sep‐Syst group, but higher in the Sep‐Port compared with the Sep‐Ctrl group (*p* < 0.01 for both, Table 1). White blood cell count (WBC) and neutrophil count decreased in all groups during the first hour of bacterial infusion, and WBC and neutrophil counts were higher in the Sep‐Port vs. Sep‐Ctrl group (*p* < 0.001 for both; Table 1). There was no difference in hemoglobin count or temperature between the groups during the bacterial infusion. Creatinine clearance was higher in the Sep‐Port compared with the Sep‐Ctrl group (*p* < 0.01). Detailed physiological data are presented in the Supporting Information (Figures S1–S12).

### 3.8 Histopathology and Immunohistochemistry

**FIGURE 5:**
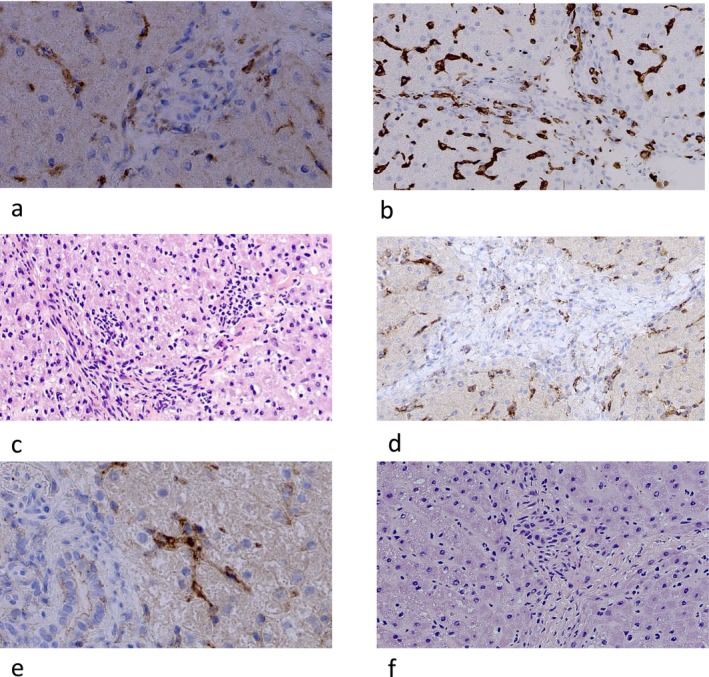
Histological evidence of inflammatory activation in the liver following *E. coli* infusion, without qualitative differences between experimental groups. Liver sections demonstrated inflammatory cell presence consistent with an early inflammatory response, while no morphologic features of overt tissue injury or infection were observed across groups. Iba1 immunohistochemistry was used to identify macrophages, including Kupffer cells, which appeared preserved irrespective of treatment. Sep‐Port and Sep‐Syst animals received budesonide via the portal vein or systemically, respectively, followed by a 3‐h portal *E. coli* infusion. Sep‐Ctrl animals received saline instead of budesonide. Representative histological and immunohistochemical features are shown and described below. (a, b): The Sep‐Port group. (a) × 40. Kupffer cells with spindle cell features show distinct Iba1 staining. The cells are positioned along the sinusoids. The portal space is visible (center). (b) × 20. Kupffer cells having irregular spindle cell features show distinct Iba1 staining. The cells are positioned along the sinusoids. The portal space with a bile duct (center) and the portal vein is visible. (c, d): The Sep‐Syst group. (c) × 20. Parts of hepatic lobules are delineated by interlobular septa. Two poorly demarcated inflammatory cell infiltrates with a predominance of neutrophils are visible in the sinusoids, and additional neutrophils are seen in the capillaries and in the vicinity of the portal space (down, center). (d) × 20. Periportal areas show strongly stained Kupffer cells lining the sinusoids. The portal space is seen in the center of the photo. (e, f): The Sep‐Ctrl group. (e) × 40. The portal space is on the left. Several strongly stained Kupffer cells lining the sinusoids are shown. (f) × 20. Portal space (center) and interlobular septum. The capillaries displayed scattered neutrophils.

Histological examination of liver tissue demonstrated inflammatory activation characterized by increased leukocytes within hepatic sinusoids, predominantly neutrophils, with occasional involvement of portal areas and interlobular septa (Figure 5). Infiltrating monocytes and macrophages were less frequent. Importantly, there was no consistent morphologic evidence of invasive infection or structural tissue injury, as liver sections generally lacked necrotic areas, abscess formation, thrombosis, or hemorrhage, and bacteria were not detected by special stains or immunohistochemistry. Only one animal in the Sep‐Ctrl group exhibited focal neutrophil aggregates resembling early microabscess formation, although no associated bacteria were identified. Immunohistochemical staining for Iba1 demonstrated preserved Kupffer cell populations in all experimental groups, with moderate to strong cytoplasmic staining and no qualitative differences between treatment groups. A more detailed description of the histological analysis can be found in the Supporting Information.

## 4 Discussion

In this experimental model of gram‐negative abdominal sepsis in pigs, hepatic bacterial elimination, assessed by calculating the hepatic to portal venous bacterial count ratio, did not differ between portal or systemic administration of budesonide. However, portal administration of budesonide enhanced the anti‐inflammatory response without increasing systemic bacterial levels, in contrast to systemic administration, which impaired bacterial elimination and resulted in higher circulating bacterial levels. In contrast, endotoxin clearance was not shown to be affected by portal or systemic budesonide administration. Budesonide administration mitigated the pro‐inflammatory response to the *E. coli* infusion compared with no such treatment, while the physiological response was dampened by both portal and systemic budesonide, with a more pronounced effect following systemic administration.

Systemic glucocorticoid administration reduced bacterial clearance in the present study. Previous experimental data on glucocorticoids and bacterial elimination are heterogeneous, likely reflecting differences in glucocorticoid compounds, dosing, timing, pathogens, and species. Glucocorticoids have been reported to both enhance and suppress phagocytic function in vitro [25, 26, 27, 28, 29], and animal models with common pathogens have similarly shown divergent effects on bacterial elimination [15, 30, 31, 32]. In our study, despite comparable portal venous bacterial counts, systemic budesonide administration resulted in higher arterial and hepatic venous bacterial levels than portal administration. This pattern suggests impaired extrahepatic bacterial elimination following systemic glucocorticoid exposure. Alternatively, hepatic bacterial elimination may have been reduced by systemic budesonide administration; however, variability in both the numerator and denominator of the hepatic to portal venous bacterial count ratio limited the ability to detect such an effect. In addition, rapid post‐infusion bacterial clearance across all groups further hindered detection of differences in elimination rates.

Previous experimental and clinical studies have demonstrated that glucocorticoids attenuate the pro‐inflammatory response in sepsis [27, 33]. We observed that the effects of budesonide on the inflammatory response differed between portal and systemic administration. Animals receiving portal budesonide exhibited a stronger anti‐inflammatory response early after bacterial infusion, while both portal and systemic glucocorticoids attenuated the pro‐inflammatory response to *E. coli* relative to untreated animals. If the liver plays a central role in coordinating anti‐inflammatory signaling and IL‐10 production, targeted hepatic exposure to budesonide after portal administration may underlie the enhanced anti‐inflammatory effect. Consistent with this interpretation, dexamethasone‐pretreated murine Kupffer cells exhibit a shift from a pro‐ to an anti‐inflammatory TLR‐4 response, characterized by reduced TNF and increased IL‐10 expression [34]. Peak levels of IL‐6, a pro‐inflammatory cytokine, were similar between groups, but cytokine concentrations declined more rapidly in glucocorticoid‐treated animals. The observed cytokine kinetics may reflect the liver's prominent contribution to systemic IL‐6 production during sepsis [12]. Alternatively, limited systemic exposure to budesonide following hepatic first‐pass metabolism may have been sufficient to dampen cytokine responses systemically. In line with previous reports, budesonide attenuated the respiratory, circulatory, and metabolic responses to the *E. coli* infusion [15, 30, 35, 36], with more pronounced effects after systemic administration, reflecting higher systemic exposure relative to portal administration and its substantial hepatic first‐pass metabolism.

Portal delivery of budesonide was associated with immunomodulation without the increase in circulating bacterial levels observed after systemic administration. In contrast, systemic budesonide treatment was associated with impaired bacterial elimination, although the precise site of this effect cannot be determined from the present data. Our results suggest that hepatic budesonide administration may allow modulation of the inflammatory response while limiting adverse effects on systemic bacterial clearance. Although direct portal venous administration is limited to experimental settings, hepatic targeting of budesonide can be achieved clinically through oral administration followed by intestinal absorption and first‐pass metabolism. Oral budesonide is currently used in the treatment of autoimmune hepatitis and is associated with fewer systemic adverse effects compared to conventional glucocorticoids [37]. As pharmacological agents can be delivered to the liver through oral administration, the concept of regional immunomodulation in sepsis merits further investigation.

### 4.1 Strengths and Limitations

To our knowledge, this is the first study to show how regional glucocorticoid treatment via the portal vein in a sepsis model can increase the acute anti‐inflammatory response without decreasing the bacterial elimination. Our large animal sepsis model has several advantages. The juvenile pig is large enough to allow instrumentation and monitoring used in the ICU, making our model clinically more relevant compared to small animal models. Moreover, the porcine liver is anatomically and physiologically similar to that of humans [38], the circulation of the pig has been suggested to be most similar to that of humans among non‐primates [39], and the porcine immune system closely resembles that of humans [40]. Also, this large animal model allows for the full complexity of the budesonide effect on bacterial elimination and systemic inflammatory response in vivo.

Nevertheless, our study has limitations. As a proof‐of‐concept study, glucocorticoids were administered before the bacterial challenge, whereas patients in septic shock receive glucocorticoids after the onset of infection. Budesonide undergoes extensive first‐pass metabolism in the liver [19], but given the relatively high budesonide dose used, some systemic effects were seen. Yet, the observed differences in bacterial elimination, systemic inflammatory response, and physiological response to the *E. coli* infusion after portal vs. systemic budesonide administration suggest that a significant amount of the initial portal budesonide dose was metabolized in the liver. Serum concentrations of budesonide or its metabolites were not measured, limiting direct assessment of systemic exposure. The glucocorticoid effects can take minutes to days to develop [41]. In our study, systemic bacterial elimination was decreased, and cytokine and physiological responses were affected 90 min after budesonide administration, indicating that enough time had passed to detect glucocorticoid effect. As an experimental proof‐of‐concept study, budesonide was administered before the bacterial challenge to enable the maximal effect of the glucocorticoid to develop. The study was conducted in conventionally raised pigs rather than specific pathogen‐free (SPF) animals, which may introduce variability related to underlying microbial exposure. All animals were clinically healthy at inclusion, showed no macroscopic abnormalities at baseline, and were evenly distributed across groups. During sampling, the abdominal cavity was inspected macroscopically in conjunction with liver and spleen biopsies, without signs of overt infection. However, a systematic post‐mortem examination was not performed; in particular, the thoracic cavity was not inspected and the lungs were not systematically sectioned to assess for abscesses or other signs of *Actinobacillus pleuropneumoniae* infection, nor was pericarditis evaluated. Consequently, the presence of subclinical infections cannot be fully excluded. The study was performed in pigs with normal microbiota, rather than specific pathogen‐free (SPF) animals.

## 5 Conclusions

In this experimental model of sepsis, hepatic bacterial elimination, assessed as the hepatic to portal venous bacterial count ratio, was preserved following portal budesonide administration and did not differ from systemic or no glucocorticoid treatment. Systemic budesonide administration was associated with impaired systemic bacterial elimination. Portal budesonide enhanced the early anti‐inflammatory response without increasing systemic bacterial levels. These findings suggest that targeted hepatic budesonide exposure may allow modulation of the inflammatory response while limiting adverse effects on systemic bacterial clearance, supporting further investigation of regional immunomodulatory strategies in sepsis.

## Author Contributions

M.L. and K.H. conceived the study. All authors contributed to the study design. K.H. performed the experiments. K.H., P.S., and F.W. conducted data collection. K.H., A.L. and E.T. performed the laboratory analyses. K.H. analyzed the data and drafted the manuscript. All authors contributed to data interpretation, critically revised the manuscript for important intellectual content, and approved the final version for publication.

## Funding

This work was supported with funding from the Uppsala University Hospital Research Fund and the Olinder‐Nielsen Foundation.

## Ethics Statement

The Uppsala Regional Animal Ethics Committee (Uppsala tingsrätt, Box 1113, 751 41 Uppsala, Sweden) approved the experiments (Dnr. C155/14 (October 29, 2014) and Dnr. 5.8.18‐08592‐/2019 (June 28, 2019)). The pigs were handled in accordance with the Guide for the Care and Use of Laboratory Animals (EU Directive 2010/63/EU).

## Consent

The authors have nothing to report.

## Conflicts of Interest

The authors declare no conflicts of interest.

## Supporting information


**Table M1** Resuscitation protocol.
**Figure M1.** Schematic illustration of the placement of the portal venous catheters for infusion and blood sampling. Two catheters were placed in the portal vein. A proximal portal venous catheter, defined as positioned a few centimeters from the liver, was used for blood sampling. A distal portal venous catheter, positioned approximately 2 cm upstream (i.e., further from the liver) relative to the proximal catheter, was used for portal infusion of *E. coli* and budesonide (the Sep‑Port group). Thus, blood sampling for bacterial cultures was performed downstream of the infusion site, immediately before entry into the liver.
**Figures S1–S10:** Time‑weighted physiological, metabolic, and hematological variables in the Portal Steroid‐Sepsis (Sep‑Port), Systemic Steroid‐Sepsis (Sep‑Syst), and Septic Control (Sep‑Ctrl) groups. Time‑weighted values were calculated over 1–4 h after the onset of E. coli infusion. Between‑group differences were assessed using ANOVA III for repeated measurements. *p < 0.05, **p < 0.01, ***p < 0.001 for differences between the Sep‐Port and Sep‐Syst groups, †p < 0.05, ††p < 0.1, †††p < 0.01 for differences between Sep‐Port and Sep‐Ctrl groups. Data are presented as mean ± SEM. BE  =  base excess; CI  =  cardiac index; MAP  =  mean arterial pressure; MPAP  =  mean pulmonary arterial pressure; WBC  =  white blood cell count.
**Figures S11** and **S12**: Urine output and creatinine clearance in the Portal Steroid‐Sepsis (Sep‐Port), Systemic Steroid‐Sepsis (Sep‐Syst) and Septic Controls (Sep‐Ctrl) groups. Between‑group differences were assessed using the Mann–Whitney U test. *p < 0.05, **p < 0.01, ***p < 0.001 for differences between the Sep‐Port and Sep‐Syst groups, †p < 0.05, ††p < 0.1, †††p < 0.01 for differences between the Sep‐Port and Sep‐Ctrl groups. Data are presented as mean  ±  SEM.

## Data Availability

Most data generated or analyzed during this study are included in this published article (and its Supporting Information files). The remaining datasets used and/or analyzed during the current study are available from the corresponding author upon reasonable request.
