# Supplementary material for: Impact of Regional Hepatic Immunosuppression With Budesonide on Bacterial Elimination in Porcine Abdominal Sepsis
Source: Acta Anaesthesiol Scand. 2026 Jul 23;70(8):e70312. doi: 10.1111/aas.70312 (PMC13395631; doi:10.1111/aas.70312)
Supplement: Supplementary file 1 — Table M1 Resuscitation protocol. Figure M1. Schematic illustration of the placement of the portal venous catheters for infusion and blood sampling. Two catheters were placed in the portal vein. A proximal portal venous catheter, defined as positioned a few centimeters from the liver, was used for blood sampling. A distal portal venous catheter, positioned approximately 2 cm upstream (i.e., further from the liver) relative to the proximal catheter, was used for portal infusion of E. coli and budesonide (the Sep‑Port group). Thus, blood sampling for bacterial cultures was performed downstream of the infusion site, immediately before entry into the liver. Figures S1–S10: Time‑weighted physiological, metabolic, and hematological variables in the Portal Steroid‐Sepsis (Sep‑Port), Systemic Steroid‐Sepsis (Sep‑Syst), and Septic Control (Sep‑Ctrl) groups. Time‑weighted values were calculated over 1–4 h after the onset of E. coli infusion. Between‑group differences were assessed using ANOVA III for repeated measurements. *p < 0.05, **p < 0.01, ***p < 0.001 for differences between the Sep‐Port and Sep‐Syst groups, †p < 0.05, ††p < 0.1, †††p < 0.01 for differences between Sep‐Port and Sep‐Ctrl groups. Data are presented as mean ± SEM. BE = base excess; CI = cardiac index; MAP = mean arterial pressure; MPAP = mean pulmonary arterial pressure; WBC = white blood cell count. Figures S11 and S12: Urine output and creatinine clearance in the Portal Steroid‐Sepsis (Sep‐Port), Systemic Steroid‐Sepsis (Sep‐Syst) and Septic Controls (Sep‐Ctrl) groups. Between‑group differences were assessed using the Mann–Whitney U test. *p < 0.05, **p < 0.01, ***p < 0.001 for differences between the Sep‐Port and Sep‐Syst groups, †p < 0.05, ††p < 0.1, †††p < 0.01 for differences between the Sep‐Port and Sep‐Ctrl groups. Data are presented as mean ± SEM. [file AAS-70-0-s001.docx]

# Supplementary Information

[Supplementary Information 1](#_Toc230851018)

[Supplementary methods and materials 2](#_Toc230851019)

[Table M1. Resuscitation protocol. 2](#_Toc230851020)

[Animals 3](#_Toc230851021)

[Anesthesia and fluid administration 3](#_Toc230851022)

[Preparatory procedure 3](#_Toc230851023)

[Organism and preparation of bacterial infusion 4](#_Toc230851024)

[Bacterial cultures from blood and organs 5](#_Toc230851025)

[Measurements 5](#_Toc230851026)

[Histopathology 6](#_Toc230851027)

[Supplementary results 6](#_Toc230851028)

[Physiologic response 6](#_Toc230851029)

[NSep-Port group 9](#_Toc230851030)

[Histopathology and immunohistochemistry 10](#_Toc230851031)

[References 10](#_Toc230851032)

## Supplementary methods and materials

### Table M1. Resuscitation protocol.

| **Parameter** | **Threshold value for intervention** | **Interventions** |
| --- | --- | --- |
| PaO_2_ | <10 kPa first time | Increase FiO_2_ to 0.45 |
| PaO_2_ | <10 kPa thereafter | 1. Increase FiO_2_ to the next level: 0.6🡪0.8 🡪1.0, and |
|  |  | 1. Increase PEEP to the next level: 5🡪8🡪10🡪14 cmH_2_O, and |
|  |  | 1. Lung recruitment maneuver^a^ |
| PaO_2_ | >20 kPa | Decrease FiO_2_ to the previous level: 1.0🡪0.8🡪0.6🡪0.45🡪0.3 |
| PaCO_2_ | >6.5 mmHg | Increase V_T_ with 10 %, to maximum 15 mL x kg^-1^ |
| PaCO_2_ | <4.5 mmHg | If respiratory rate is ≤25, decrease V_T_ with 10 %. If respiratory rate is >25, decrease it with 10 %. |
| MAP (<60 min) | MAP=MPAP (<60 min after start of *E. coli*-infusion) | Single dose of 40 µg norepinephrine i.v. |
|  | MAP<50 mmHg (<60 min after start of *E. coli*-infusion) | Start norepinephrine infusion (20 µg x mL^-1^) 5 mL x h^-1^. If ongoing norepinephrine infusion, increase rate one step^b^. |
| MAP (>60 min) | MAP<60 mmHg (>60 min after start of *E. coli* infusion) | Start norepinephrine infusion (20 µg x mL^-1^) 5 mL x h^-1^. If ongoing norepinephrine infusion, increase rate one step^b^. |
| MAP | MAP=MPAP (>60 min after start of *E. coli* infusion) | 1. Single dose of 20 µg norepinephrine i.v., and 2. Start norepinephrine infusion (20 µg x mL^-1^) 5 mL x h^-1^. If ongoing norepinephrine infusion, increase rate one step^b^, and 3. Fluid bolus with acetated Ringer’s solution 15 mL x kg^-1^ |
| MAP | MAP>100 mmHg | If ongoing norepinephrine infusion (20 µg x mL^-1^), decrease rate one step: 40🡪20🡪10🡪5🡪0 mL x h^-1^ |
| CO | CO<2.0 L x min^-1^ | Fluid bolus with acetated Ringer’s solution 15 mL x kg^-1^ and/or start norepinephrine infusion (20 µg x mL^-1^) 5 mL x h^-1^. If ongoing norepinephrine infusion, increase rate one step^b^. |
| Blood Glucose | <4.0 mmol x L^-1^ | Give 20 mL of 30 % glucose-solution i.v. |

^a^ PEEP was increased stepwise until a peak pressure of 35 cm H_2_O was reached. Then, an inspiratory hold was performed for 10 s. Thereafter, the PEEP was stepwise decreased to the PEEP defined by the protocol. If MAP decreased to the level of the MPAP, the recruitment maneuver was aborted.

^b^ Norepinephrine infusion increased stepwise: 5🡪10🡪20🡪40 mL x h^-1^.

Abbreviations: CO=cardiac output, *E. coli* = *Escherichia coli*, FiO_2_ = inspired fraction of oxygen, I:E = Inspiratory:Expiratory ratio, MAP = mean arterial pressure, MPAP = mean pulmonary arterial pressure, PaCO_2_ = arterial partial pressure of carbon dioxide, PaO_2_ = arterial partial pressure of oxygen, PEEP = positive end expiratory pressure, RR = respiratory rate, V_T_ = tidal volume.

### Animals

The pigs were housed in a farm and transported to the laboratory prior to the experiments. Animals had access to water and food ad libitum until 1 h before the start of the experiment.

### Anesthesia and fluid administration

On arrival, general anesthesia was induced by injecting a mixture of tilétamin-zolazepam 6 mg x kg^-1^ and xylazin 2.2 mg x kg^-1^ intramuscularly. Anesthesia was maintained with sodium pentobarbital 8 mg x kg^-1^ x h^-1^ and morphine 0.26 mg x kg^-1^ x h^-1^ dissolved in 2.5 % glucose given as a continuous infusion. Rocuronium bromide 2.5 mg x kg^-1^ x h^-1^ was continuously administered to prevent shivering, and the dose was adjusted if shivering was detected. Acetated Ringer’s solution was administered as an initial bolus of 20 mL x kg^-1^ over two hours following anesthesia induction, adjusted by subtracting 100 mL to account for the volume of budesonide or saline infusion, and continued at 2 mL x kg^-1^ x h^-1^ thereafter. This resulted in a total basal fluid administration rate of 10 mL x kg^-1^ x h^-1^.

### Preparatory procedure

All preparations were performed under aseptic conditions. The airway was secured by tracheotomy and the pigs were mechanically ventilated throughout the experiment using a Servo I ventilator (Maquet Critical Care, Stockholm, Sweden). A cervical artery was catheterized for pressure monitoring and blood sampling. A central venous line and a Swan-Ganz catheter were inserted through the right external jugular vein into the superior caval vein and into the pulmonary artery, respectively. Two 4F single-lumen catheters were placed in the portal vein through the splenic vein via left subcostal incision (Figure M1). A proximal portal venous catheter, positioned a few centimeters from the liver, was used for blood sampling. A distal portal venous catheter, positioned approximately 2 cm upstream (i.e., further from the liver) relative to the proximal catheter, was used for portal infusion of *E. coli* and budesonide (the Sep‑Port group). A 7Fr catheter was inserted to a hepatic vein through the left external jugular vein. The location of the hepatic and portal venous catheters was controlled using fluoroscopy. A urinary catheter was inserted into the bladder by performing a minimal vesicotomy. The animals were covered, and a heating pad (Operatherm 200W; KanMed, Bromma, Sweden) was set to 38°C to decrease heat losses, and turned off after six hours or if the animals' core temperature reached 42°C. After completed preparations, the animals were placed on their right side.


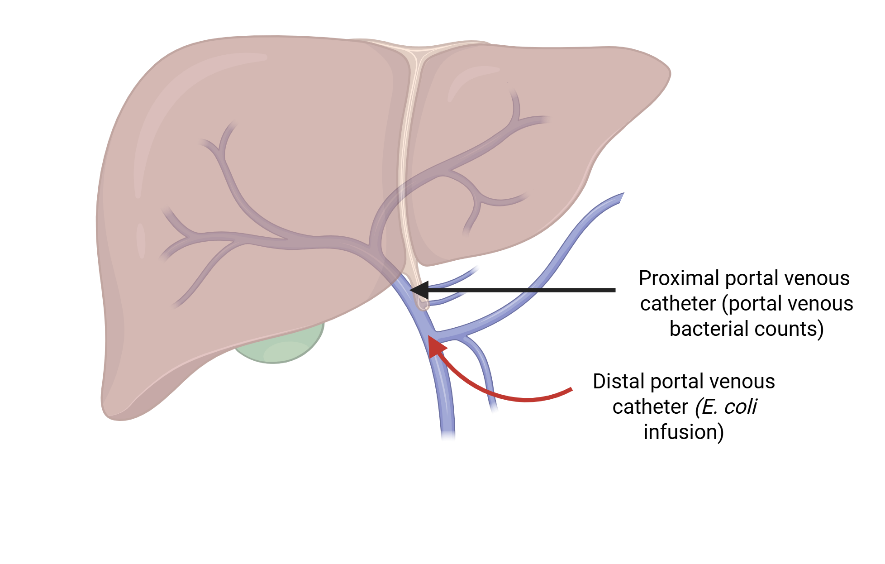


Figure M1. Schematic illustration of the placement of the portal venous catheters for infusion and blood sampling. Two catheters were placed in the portal vein. A proximal portal venous catheter, defined as positioned a few centimeters from the liver, was used for blood sampling. A distal portal venous catheter, positioned approximately 2 cm upstream (i.e., further from the liver) relative to the proximal catheter, was used for portal infusion of *E. coli* and budesonide (the Sep‑Port group). Thus, blood sampling for bacterial cultures was performed downstream of the infusion site, immediately before entry into the liver.

### Organism and preparation of bacterial infusion

The E. coli (B09-11822 serotype O-rough:K1:H7; Statens Seruminstitut, Copenhagen, Denmark) used in the experiment is an encapsulated clinical isolate. Before the experiment, the *E. coli* was harvested from a frozen isolate, reinoculated on a cysteine lactose electrolyte deficient (CLED) plate and cultured overnight in an incubation chamber at 37 ^o^C. The *E. coli* were grown to logarithmic growth phase in lysogeny broth medium according to Miller (LB) (VWR, Spånga, Sweden) two hours before the start of the experiment. The LB broth and bacteria suspension were then centrifuged at 6000 rpm at 22°C for two minutes to enable LB removal. The remaining bacteria were resuspended in phosphate-buffered saline (PBS). Concentration of the bacterial suspension was assessed using spectrophotometry and adjusted when needed.

A target bacterial dose of a total of 8.23 log_10_ colony-forming units (CFUs) in 20 mL saline was infused in the portal vein during three hours. Fresh subcultures were prepared and grown into logarithmic growth phase before each experiment, and to secure that the bacteria remained in logarithmic growth phase, the infusate was replaced hourly. The number of bacteria in the infusate was controlled after completed bacterial infusion.

### Bacterial cultures from blood and organs

Bacterial counts were determined by plating 0.1 mL blood in triplicate on CLED plates from the portal vein, hepatic vein and artery. Biopsies for bacterial counts were obtained from standardized localizations in the right lobe of the liver and the spleen under aseptic conditions immediately after sacrificing the animals. Biopsies of 1–2 g obtained were placed in 3 mL saline and homogenized using sterile glass vessel tissue grinders of Potter-Elvehjem type (VWR, Spånga, Sweden) and 0.2 mL of each solution was plated in triplicate on CLED plates to determine viable count. The detection limit for the growth of bacteria was 5 CFU x mL^-1^. The bacterial counts were corrected for variations in pig size and differences in infused bacterial dose. E. coli was identified by colony morphology.

### Measurements

Mean arterial pressure (MAP), mean pulmonary arterial pressure (MPAP), heart rate (HR), and central venous pressure (CVP) were continuously monitored using a Philips IntelliVue MX800 or MP50 patient monitor (Philips Healthcare, Eindhoven, The Netherlands). Cardiac output (CO) was measured using a Swan–Ganz catheter. Urine output was measured, and urine samples were collected once before baseline and once from pooled urine collected over the 0-4 hour period. Airway pressure values and respiratory volumes were recorded from ventilator readings. Cytokine concentrations were measured using porcine‑specific sandwich enzyme‑linked immunosorbent assays (ELISA; DY690B for TNF‑α, DY686 for IL‑6, and DY693B for IL‑10; R&D Systems, Minneapolis, MN, USA). Blood from a cervical artery was analyzed for pH, gas tensions (PaO₂, PaCO₂), oxygen saturation, lactate, base excess, and hemoglobin using an ABL™ 800 and a Hemoximeter™ OSM‑3 (Radiometer, Brønhøj, Denmark). The SOFA score [1] was used to assess the presence of sepsis‑related organ dysfunction based on standard parameters: respiratory function (PaO₂/FiO₂ ratio), coagulation (platelet count), cardiovascular status (MAP and need for vasoactive support), and renal function (creatinine and urine output). The central nervous system component was not evaluated due to sedation, and bilirubin was not measured during the experiment.

### Histopathology

We performed histological studies to assess the extent of inflammation in the liver, including immunohistochemistry to evaluate the presence of Kupffer cells and bacteria. Liver specimens were collected post mortem and initially stored in -70°C. 2-3 specimens from each group were randomly selected for histopathological and immunohistochemistry analysis. The samples were analyzed by a veterinary pathologist. For histopathology, a microscope Nikon (mod. Eclipse E600, Japan) was used. Upon analysis, the specimens were fixed in 4% formaldehyde and embedded in paraffin. The specimens were then cut to 4 µm slices, transferred to glass slides, and stained with hematoxylin and eosin, May-Grunwald Giemsa, and Gram stains for histopathological analysis and identification of bacteria. Stained sections were examined by light microscopy. Hepatic architecture, degree of necrosis, inflammatory infiltrates and microabscesses were assessed semi-quantitatively. Immunohistochemistry (IHC) was performed using antibodies against Iba-1 to visualize Kupffer cells.

## Supplementary results

### Physiologic response


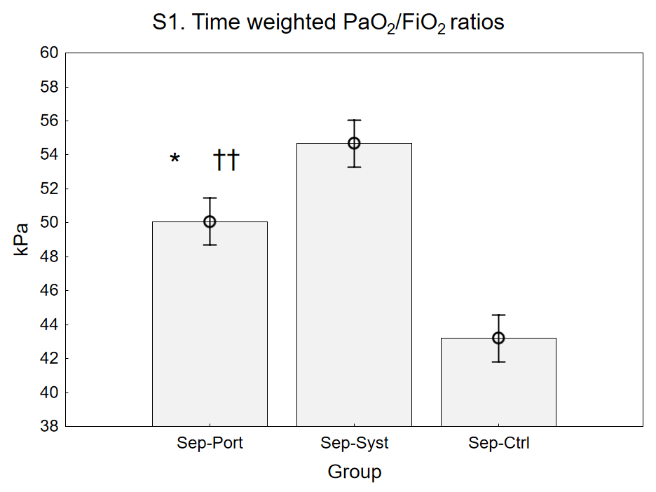

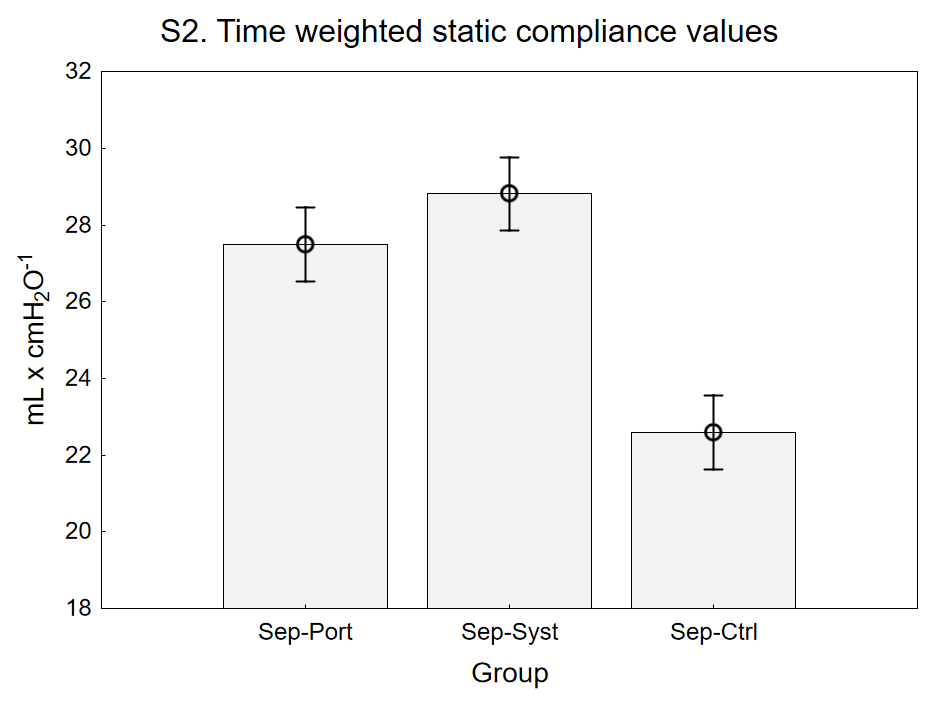

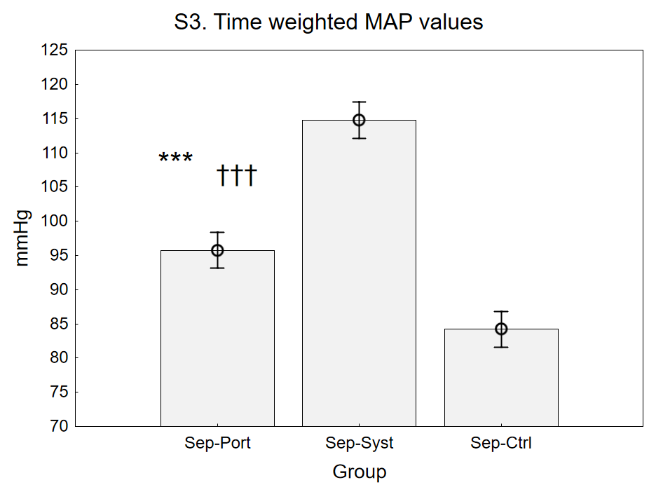

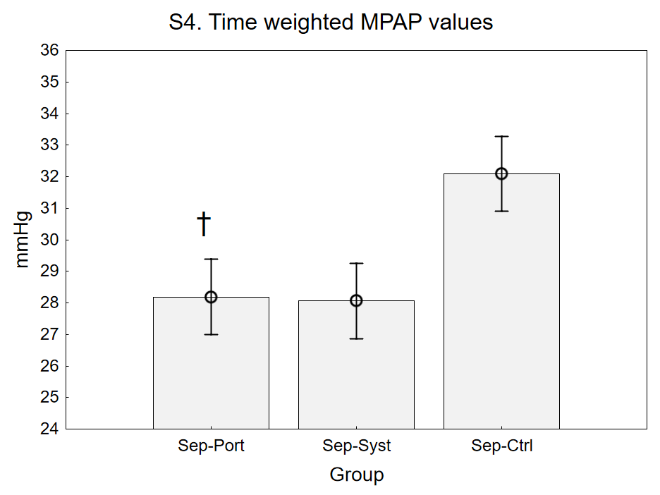

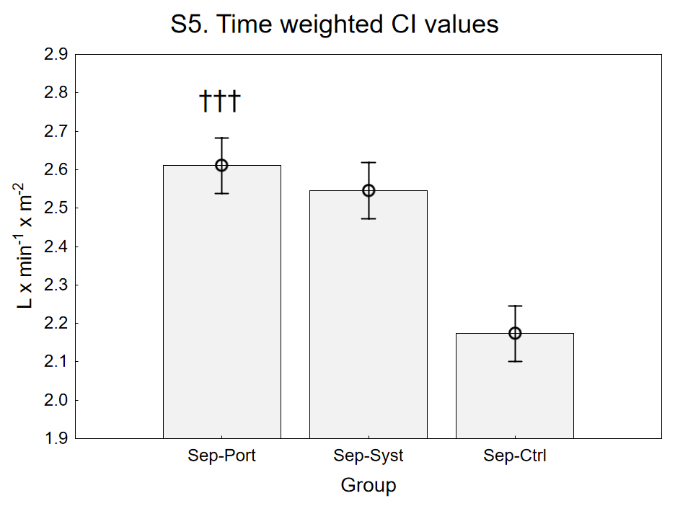

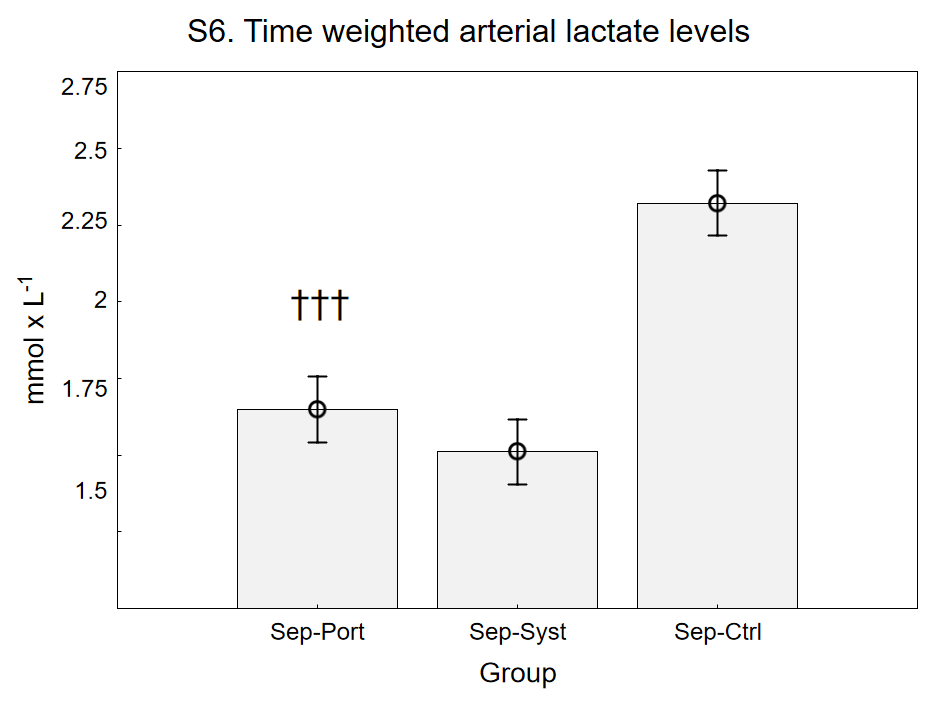

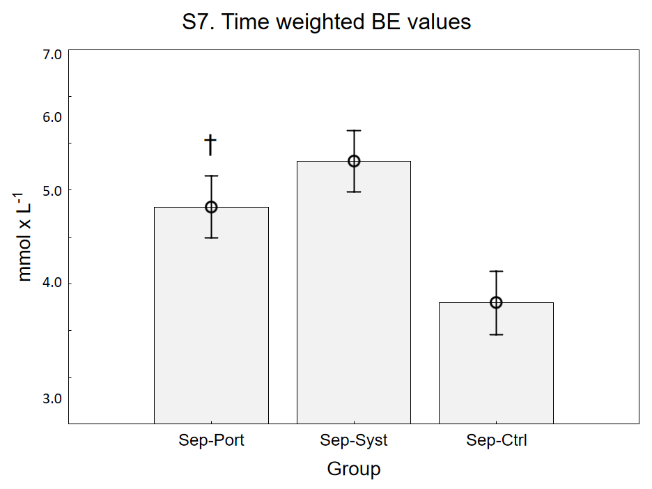

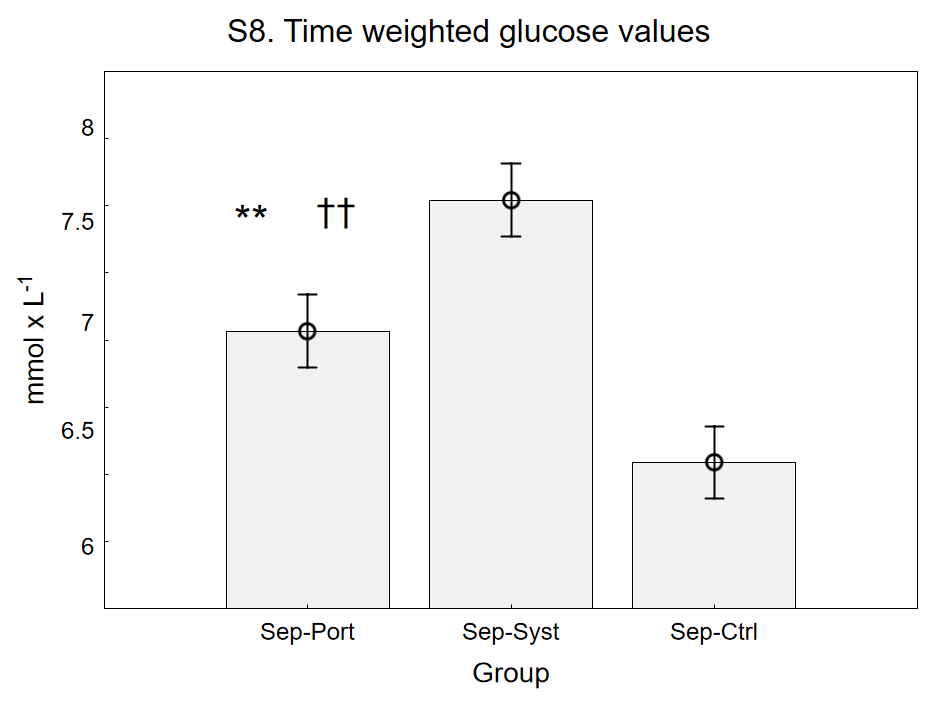

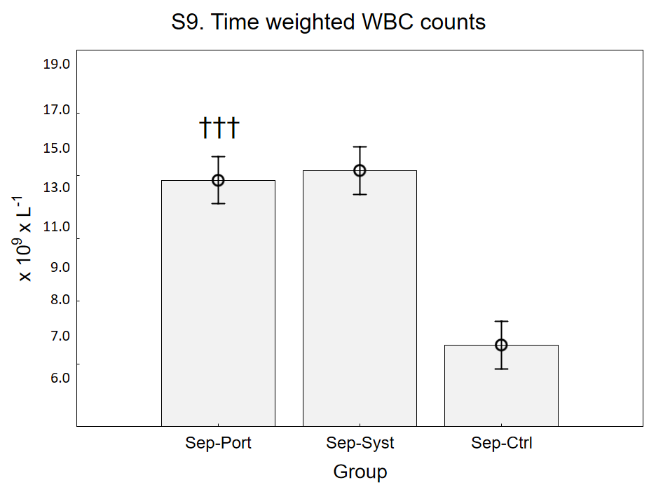

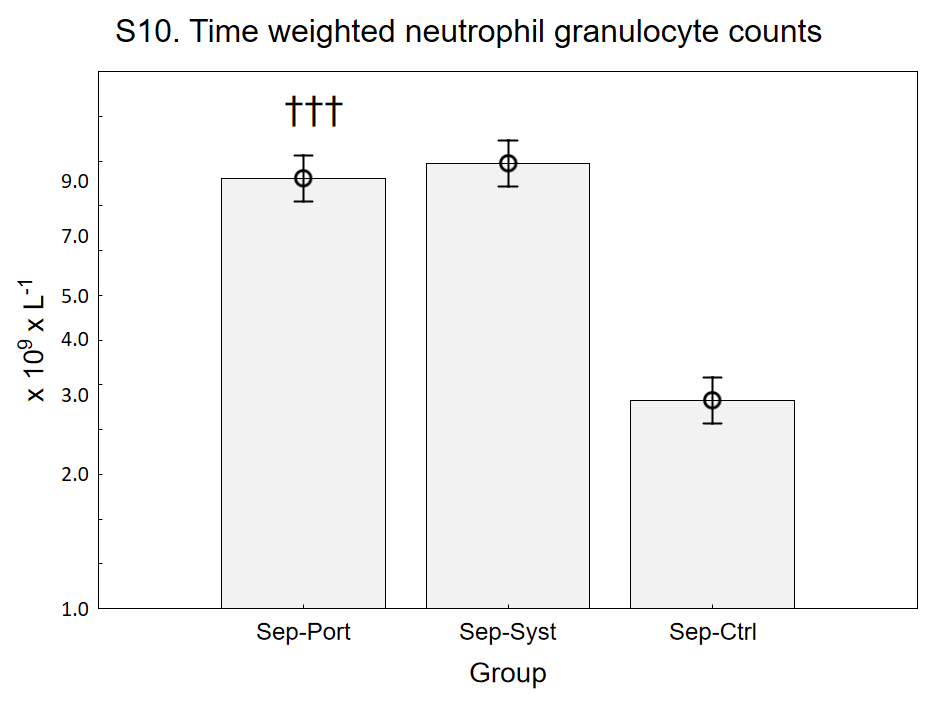


Figures S1-S10. Time‑weighted physiological, metabolic, and hematological variables in the Portal Steroid-Sepsis (Sep‑Port), Systemic Steroid-Sepsis (Sep‑Syst), and Septic Control (Sep‑Ctrl) groups. Time‑weighted values were calculated over 1–4 hours after the onset of *E. coli* infusion. Between‑group differences were assessed using ANOVA III for repeated measurements. *p<0.05, **p<0.01, ***p<0.001 for differences between the Sep-Port and Sep-Syst groups, †p<0.05, ††p<0.1, †††p<0.01 for differences between Sep-Port and Sep-Ctrl groups. Data are presented as mean ± SEM. BE = base excess; CI = cardiac index; MAP = mean arterial pressure; MPAP = mean pulmonary arterial pressure; WBC = white blood cell count.


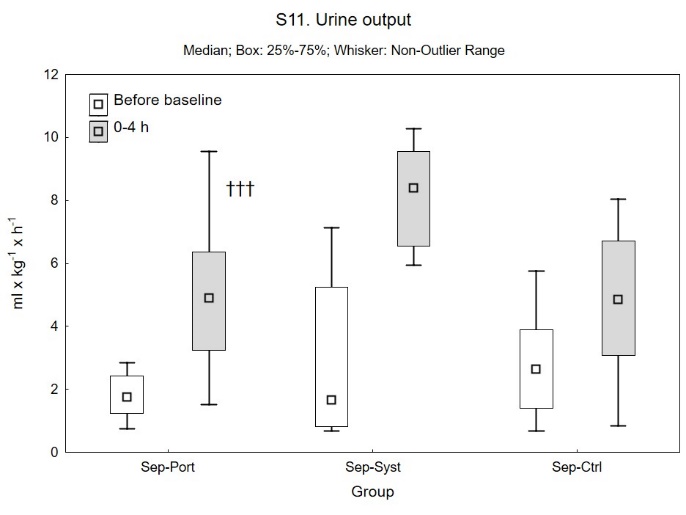

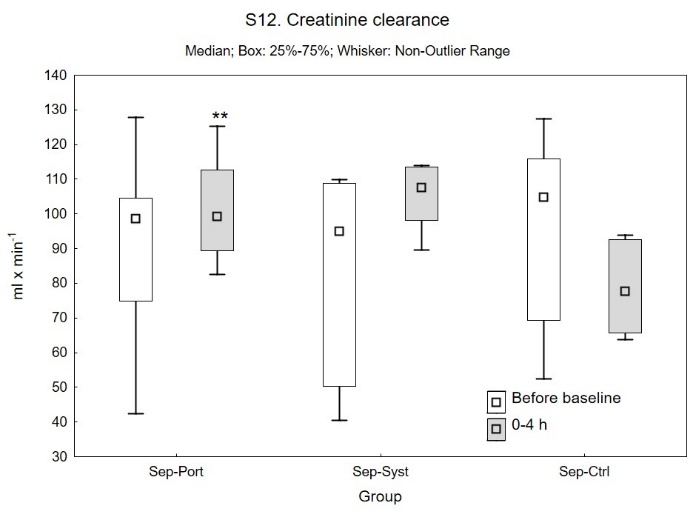


Figures S11-12. Urine output and creatinine clearance in the Portal Steroid-Sepsis (Sep-Port), Systemic Steroid-Sepsis (Sep-Syst) and Septic Controls (Sep-Ctrl) groups. Between‑group differences were assessed using the Mann-Whitney U test. *p<0.05, **p<0.01, ***p<0.001 for differences between the Sep-Port and Sep-Syst groups, †p<0.05, ††p<0.1, †††p<0.01 for differences between the Sep-Port and Sep-Ctrl groups. Data are presented as mean ± SEM.

### NSep-Port group

One animal in the NSep-Port group developed circulatory instability and received noradrenaline infusion after the start of the budesonide infusion. The animal stabilized and no longer needed noradrenaline infusion at baseline. None of the other animals receiving budesonide developed respiratory or circulatory instability when the drug was administered.

### Histopathology and immunohistochemistry

The sinusoids in some livers showed increased leukocytes with a predominance of neutrophils (PMN), a possible indication of an early inflammatory cell reaction or an early stage of infection. PMN were also seen in the portal areas and in occasional interlobular septa. Infiltrated monocytes and macrophages were less numerous. Occasional livers displayed pigment-laden macrophages or macrophages carrying small cytoplasmic granules in portal areas and interlobular septa. The pigment could consist of lipofuscin, not necessarily related to infection or inflammation. Scattered between hepatocytes, low numbers of damaged hepatocytes and cells showing chromatin margination, nuclear fragmentation or condensed nuclei suggestive of necrosis were also observed. Only one animal, in the Sep-Ctrl group, displayed numerous PMN in aggregates resembling early microabscesses. However, neither the special stains nor immunohistochemistry (IHC) revealed bacteria associated with these lesions. Apart from intravascular PMN singly and in aggregates, and the liver in the Sep-Ctrl group named above, the liver sections did not show necrotic areas, abscesses, thrombosis, hemorrhages, or other lesions consistent with an infection. Bacteria were not observed using the May-Grünwald Giemsa and Gram stains. Sporadic sections displayed a few, scattered, poorly stained rods or granules, but their identity could not be determined. Iba1 staining, used for identifying macrophages, including Kupffer cells, was observed in the cytoplasm of cells lining the capillaries, consistent with Kupffer cells, and also some endothelial cells. Scattered mononuclear cells suggestive of macrophages in the portal areas and interlobular septa were also stained. In all sections, irrespective of the experimental treatment, the Iba1-stained cells appeared relatively well preserved, showing a moderate to strong cytoplasmic staining. However, in general, the HIC-staining was deemed as sub-optimal due to the background staining in the surrounding cells. No differences between the Sep-Port, Sep-Syst and Sep-Ctrl were observed.

## References

1. Vincent, J.L., et al., *The SOFA (Sepsis-related Organ Failure Assessment) score to describe organ dysfunction/failure. On behalf of the Working Group on Sepsis-Related Problems of the European Society of Intensive Care Medicine.* Intensive Care Med, 1996. **22**(7): p. 707-10.
